# Problem alcohol use among problem drug users in primary care: a qualitative study of what patients think about screening and treatment

**DOI:** 10.1186/1471-2296-14-98

**Published:** 2013-07-13

**Authors:** Catherine Anne Field, Jan Klimas, Joseph Barry, Gerard Bury, Eamon Keenan, Bobby P Smyth, Walter Cullen

**Affiliations:** 1 UCD School of Medicine and Medical Science, Coombe Healthcare Centre, Dolphins Barn, Dublin 8, Ireland; 2 Department of Public Health and Primary Care, Trinity College Dublin, Dublin, Ireland; 3 Department of Public Health, Health Services Executive, Dublin, Ireland; 4 Addiction Services, Health Services Executive, Dublin, Ireland; 5 Graduate Entry Medical School, Faculty of Education & Health Sciences, University of Limerick, Limerick, Ireland

**Keywords:** Alcohol, Brief intervention, Illicit drugs, Primary health care, Opioids, Qualitative interviews, Screening, Methadone

## Abstract

**Background:**

Problem alcohol use is common and associated with considerable adverse outcomes among patients who attend primary care in Ireland and other European countries for opiate substitution treatment. This paper aims to describe patients’ experience of, and attitude towards, screening and therapeutic interventions for problem alcohol use in primary care.

**Methods:**

This qualitative study recruited problem drug users (N = 28) from primary care based methadone programmes in the Ireland’s Eastern region, using a stratified sampling matrix to include size of general practice and geographical area. Semi-structured interviews were conducted and analysed using thematic analysis, and audited by a third reviewer.

**Results:**

We identified three overarching themes relevant to the purpose of this paper: (1) patients’ experience of, and (2) attitude towards, screening and treatment for problem alcohol use in primary care, as well as their (3) views on service improvement. While most patients reported being screened for problem alcohol use at initial assessment, few recalled routine screening or treatment. Among the barriers and enablers to screening and treatment, patients highlighted the importance of the practitioner-patient relationship in helping them address the issue. Nevertheless, patients felt that healthcare professionals should be more proactive in the management of problem alcohol use at a primary care level and that primary care can play an important role in their treatment.

**Conclusions:**

Problem alcohol use is an important challenge in the care of problem drug users. While primary care is well placed to address this issue, little data has reported on this topic. The development of interventions which promote screening and brief interventions in practice are likely to benefit this at-risk group and further research and education, that help achieve this goal, are a priority. Strategies such as dissemination of clinical guidelines, educational videos, academic detailing and practice visits, should be explored.

## Background

Problem alcohol use is common among problem drug users attending primary [1] and secondary-care [2-4]. It is associated with serious health implications and impacts negatively on many health issues that commonly affect problem drug users, e.g. chronic hepatitis C infection [5], increased risk of fatal opiate overdose [6], compromised metabolism of methadone [7,8], among others.

Evidence describing interventions for this issue in problem drug users is scarce [9-11], and when it comes to primary care, non-existent [12]. In the absence of this evidence, we can draw some implications for care of problem drug users from the interventions developed for this problem in the general adult population. Indeed, early therapeutic interventions, such as screening and brief intervention, are effective in primary care [13]. Though they can reduce the extent of problem alcohol use by 10-35% [14,15], they are not routinely implemented in primary care [16-18]. Furthermore, their positive effect was confirmed by a clinical trial among problem drug users conducted in a specialist, secondary-care clinic [19], which suggests that if they are applied to this group attending primary care, they may work for them as well.

GPs’ views about the barriers to implementing this evidence in primary care have been explored extensively [20]. The sensitive nature of the problem, awareness of a patient's alcohol problem, patient factors, availability of intervention tools, an expectation that interventions will have limited effectiveness, lack of time, limited prior training in screening and treating problem alcohol use and fear of a negative response from patients patient have all been identified as reasons why healthcare professionals do not screen for and treat problem alcohol use [21,22].

Patients’ views on this matter can not only deepen our understanding of the barriers or enablers to screening and brief intervention, but can also help us to develop interventions that are specific to their needs [23]. Patients views are particularly important as they may be at odds with those of healthcare professionals, for example which healthcare professional is best placed to address the issue [24] and whether GPs are sufficiently confident to address problem alcohol use [25].

However, the views of drug users attending primary care are missing which further emphasizes a need for more research in this setting. It is likely that their experience may be different as they represent a high-risk group with special health-care needs and opinions [26]. For example, a qualitative study conducted in the secondary care illuminated how specific these needs could be. Problem drug users receiving methadone treatment in an addiction clinic reported that attitudes of healthcare providers were critical factors in engaging them with general medical and chronic care treatment, and also stressed the importance of various other forms of support and personal motivation [27,28]. As identified by Nyamathi et al., authors of the cited qualitative study, these barriers were similar to factors which prevented seeking help from health institutions in the first place.

To summarize the evidence, or lack thereof, on the use of alcohol screening and brief interventions in populations attending primary care, it is clear that screening and brief intervention improves health outcomes associated with problem alcohol use in the general population (and this is supported by patients’ views too), however, further research is needed among high-risk patient groups, especially problem drug users [12].

To date, the issue of screening and treatment for problem alcohol use among problem drug users attending primary care has not been explored from patients’ perspective. In particular, documenting their experience of, and identifying the potential barriers/enablers to, these interventions is necessary to inform integration of addiction treatment into primary care.

Since 2009, our group has been working on mixed methods study which aims to improve the care of problem alcohol use among patients with opiate dependency by:

– describing the experience of (and attitudes towards) screening and treatment for problem alcohol use among methadone users (Phase 1);

– developing a complex intervention, including clinical guidelines, to improve screening and treatment rates (Phase 2);

– determining the views of professionals and patients regarding optimum implementation of this complex intervention (Phase 3).

The outputs of this work to date include:

Phase 1: Qualitative interviews with 68 healthcare professionals and patients at 23 purposively sampled GP practices and specialist addiction services about their experience and attitudes towards screening and treatment for problem alcohol use among patients on methadone indicated that ‘professional education and training’ and a lack of ‘specialist support staff’ are key structural barriers hindering the implementation of alcohol BIs among GPs [29].

Phase 2: Clinical guidelines for screening and treatment for problem alcohol use among problem drug users, informed by the findings of interviews conducted in Phase 1, expert opinion through a Delphi-facilitated expert consensus process and a Cochrane Systematic Review [11], were completed in 2012 [30].

Phase 3: A cross sectional survey of 202 GPs providing methadone treatment in Ireland, documented their practice of and attitudes towards the management of problem alcohol use among methadone patients: 75% of GPs reported screening for problem alcohol use, 52% reported discussing the risks of problem alcohol use with patients, 49% performed a brief intervention and 27% referred patients to specialist services [31]. Education and training in addiction-related care was considered the most important barrier to the effective management of problem alcohol use, followed by poor service availability and the attitude of the patient.

The current paper reports on the results of the first phase of this research programme which aimed to describe patients’ (1) experience of, and (2) attitude towards, screening and therapeutic interventions for problem alcohol use in primary care. Our key question was: *What do patients think about screening and treatment?*

## Methods

### Setting

Primary care in Eastern Region of Ireland, where most problem drug users in Ireland attend for treatment (85% of national total) [32]. For the purpose of this study, we consider ‘primary care’ to consist of general practices (GP) that prescribe methadone and addiction treatment clinics that are based in a community and where general practitioners are responsible for clinical patient care.

In Ireland, to prescribe methadone, GPs are subject to clinical audit and must complete special training, with GPs providing treatment for 15 or more patients subject to more regular audit and advanced training. GPs who prescribe methadone for less than 15 patients are referred to as ‘level one GPs,’ and those prescribing for 15 or more as ‘level two’ GPs. Therefore, these are considerably different groups with different training and competency levels. Initiation of methadone therapy, treatment of patients with more complex medical and psychosocial needs (including alcohol dependence) and unstable drug use is only permitted by specialist addiction treatment services or by ‘level two’ GPs. In 2009, there were 277 GPs (Level 1 = 218) in Ireland prescribing methadone to 3200 patients, out of 8551 patients being prescribed methadone nationally [32].

### Participants

Problem drug users were recruited by their prescribing GPs. At the outset, all GPs in the Eastern Region, who were registered on the Central Treatment List^a^, were invited to take part in the study (n = 150). At the time of the study, this region was divided in three areas, Northern, South-West and East Coast for the purposes of planning and delivery of addiction services. A purposive sampling framework, which included geographical region and primary care agency as the sampling parameters, was used to recruit equivalent numbers of patients for each parameter. Table 1 summarizes socio-demographic and addiction characteristics of the sample.

**Table 1:** Socio-demographic and addiction characteristics

	
Male	21
Mean Age (SD)	39.4 (8.2)
Unemployed	24
Attends Level 1 GP	12
Attends Level 2 GP	16
*Geographical area of GP:*	
Southwest	17
East	2
North	9
*Housing:*	
Council house	8
Rented	12
Owned	4
Transitional	1
Parents’ house (family)	1
*Past Drug use:*	
Heroin	23
Cocaine (crack)	2
Codeine (opiates)	1
Speed (amphetamines)	1
Mean Age of onset (SD)	20.6 (7.0)
Ever injected drugs	23
Mean age of first injection (SD)	21.5 (7.0)
*Current Drug use:*	Yes = 15
Heroin	4
Cocaine (crack)	1
Cannabis	6
Benzodiazepines	6
Currently injecting	5
Hepatitis C positive (HCV)	16
Mean length of methadone use in years (SD)	14.1 (8.2)
Current mean methadone dose (SD)	75.4 (23.8)
Mean AUDIT score (SD)^*^	11 (9.6)
Low-risk drinking	9
Hazardous drinking	8
Harmful drinking	0
Dependent drinking	5
Missing information	6

^*^ We used AUDIT in a preceding prevalence survey in this population [1], and considered it a valid tool to establish level of problem alcohol use (i.e. low-risk drinking = 0–7, hazardous = 8–16, harmful = 16–19, dependent = 20+).

Thirty-seven GPs/practices expressed an interest in participating. Potential participants were allocated to a sampling matrix and a quota randomly sampled from each cell. Selected GPs (n = 20) were contacted and given information on the study, its aims, recruitment, participant information and consenting procedures. Each GP was asked to recruit 2–3 patients based on the following selection criteria:

Patient selection criteria:

• Aged ≥18 years.

• Current alcohol use.

• No language difficulties.

• No severe mental health problems.

Recruitment took place at eight practices in the Northern Area, eight in the South West Area and four in the East Coast Area, and two addiction clinics (reflecting the distribution of GPs and service organisation) [33].

### Data collection

Semi-structured interviews were conducted with 28 patients (mean duration = 28 minutes) between July 2010 – January 2011. The final decision on sample size was based on evidence that data saturation had been reached [33]. The interview followed a topic guide which was informed by a literature review [12] and pilot study and consisted of three areas:

• *Demography and descriptive data*, including the AUDIT screening questionnaire (Alcohol Use Disorders Identification Test) [34].

• *Experience* of screening/treatment for problem alcohol use.

• *Attitude* towards screening/treatment for problem alcohol use.

### Data analysis

Thematic analysis followed a five-step process outlined by Braun and Clarke [35]: 1) Preparing data: transcribing, and familiarising yourself with your data; 2) Generating initial codes; 3) Searching for the themes; 4) Reviewing the themes; 5) Defining and naming themes. This procedure was first conducted by two independent coders (CAF, JK) on three interviews and then repeated by one coder (CAF) for the remaining transcripts. Themes were audited by a third reviewer (WC) and facilitated by a qualitative software package NVivo 8 (http://www.qsrinternational.com).

### Ethical considerations

The Irish College of General Practitioners Research Ethics Committee approved this study (September 17th, 2008). Research carried out on humans in this study is in compliance with the Helsinki Declaration (http://www.wma.net/en/30publications/10policies/b3/index.html). This study adheres to the RATS guidelines on qualitative research (http://www.biomedcentral.com/ifora/rats).

Potential participants were provided with information regarding the study by their GP and, if willing to participate, an appointment to meet with a researcher. At this meeting, patients were provided with further explanation on the study, the nature of the questions that would be asked and they were encouraged to express any concerns or issues requiring clarification. In particular, it was made explicit to patients that non-participation in the study would not compromise the care they receive. Participation in the study was on a voluntary basis and no inducements to participate were offered.

When all such issues had been explained to the patients’ satisfaction, they were asked to consent to participate in the study by signing a consent form. While information from individual interviews was not reported to the patients’ GP, all patients were advised to discuss the issue of problem alcohol use and any issues that had been raised by the interview with their GP. All data was anonymised and any details that could potentially identify individuals were removed.

## Results

### Socio-demographic and addiction characteristics

Twenty eight interviews were recorded (21 male, mean age 39.4 years [SD = 8.2], 24 unemployed, 20 living in social housing/rental accommodation. Table 1 below summarizes socio-demographic information.

With respect to the addiction characteristics of the sample, heroin was reported as a main drug of choice for a majority of patients (23), with an average age of first use as 20.6 years (SD = 7). As identified by the AUDIT screening questionnaire, nine patients fell into the low-risk drinking category, eight into hazardous drinking category and five into dependent drinking category. Further addiction characteristics can be found in the Table 1.

### Thematic analysis

Eight main themes and 19 sub-themes were identified. In this paper, we present data for the three of these themes relevant to the purpose of this paper. They are: (1) patients’ experience of, and (2) attitude towards, screening and treatment for problem alcohol use in primary care, as well as their (3) views on service improvement. See the ‘Summary of Main Themes and Subthemes’ section.

1) Patients’ experience of therapeutic interventions for problem alcohol use.

a) Patients’ experience of being screened for problem alcohol use.

The majority of patients were able to recall being screened at some stage. Patients, who reported no screening, explained that their healthcare professionals were not concerned about problem alcohol use as there was an understanding that it was not an issue for that patient. For those, who were screened for alcohol use in the past, the most common time of screening was at initial assessment, with most reporting no screening thereafter, except if there was a suspicion of problem alcohol use. In terms of screening methods, patients simply described “being asked” about their alcohol use, they did not report being subject to structured screening such as the ‘AUDIT’, nor did they recall being breathalysed or having liver function tests (LFTs). In some cases, patients themselves told the healthcare professional about their alcohol use:

a) Patients’ experience of interventions for problem alcohol use.

Patients were asked had they ever received any advice or ‘intervention’ about their alcohol use, specifically any form of advice or counselling that the healthcare professional may have provided. A number of patients reported receiving some form of what could be best described as a ‘brief intervention’, i.e. to give advice and motivate the patients to either alter their drinking habits or seek treatment, if needed [36]. Patients explained how healthcare professionals gave them advice about the negative effects of problem alcohol use and also gave them advice about cutting down, drinking in moderation and possibly seeking further help.

The main advice that patients received from the healthcare professionals was about the negative effects of problem alcohol use. The most common form of advice was warning about the hepatic effects of problem alcohol use (e.g. how problem alcohol use exacerbates the risk of liver damage by the presence of Hepatitis C -HCV). Other advice included reducing their alcohol intake and aiming to drink in moderation.

A number of patients reported receiving medication for problem alcohol use; however, this was not as common as the brief intervention or advice. Three patients had received an outpatient detoxification with chloradiazepoxide (‘Librium’ ®) and had found it useful at the time. However, all noted that the detoxification was ‘too short’. Although they did not drink when they were taking it, they subsequently drank when the week was complete.

A number of patients reported being referred to specialist psycho-social services, including psychiatry and counselling services. Referral to counselling was often for multiple issues other than alcohol, such as addiction to benzodiazepines or personal and social problems. Most recalled a positive experience of counselling and reported that they were able to discuss problem alcohol use with counsellors.

Restrictions imposed by healthcare professionals, as a way of tackling problem alcohol use were also reported, although patients acknowledged that this was more common in addiction clinics. Negative consequences included increased supervision, breathalysing, delayed dispensing and supervised consumption of methadone. Patients acknowledged that, in some cases, it is a necessary procedure due to the risks attached to problem alcohol use among methadone users.

2) Patients’ attitudes to therapeutic interventions

Patients’ attitudes to therapeutic interventions varied greatly; while some did not have an issue being asked about problem alcohol use or receiving advice, others acknowledged that although they did not like it they understood that it was necessary.

At the other end of the spectrum, some patients described their annoyance at being questioned, and others were able to identify that they became defensive and, at times, concealed their alcohol use.

a) Acceptance of therapeutic interventions

A positive or accepting attitude to therapeutic interventions was reported by a number of patients. Some patients initiated the discussion on alcohol use with their healthcare professional because it was a concern to them, but also because they felt happy to discuss it. Similarly, there were patients who did not mind discussing it, as they felt they had “nothing to hide”. There were other patients, who reported being receptive to the advice they received, such as the patient below, who described being happy that the healthcare professional was concerned for him.

a) Negative reactions to therapeutic interventions

Negative reactions included fear, embarrassment and resentment. Some patients did not feel comfortable discussing their problem alcohol use. Others were afraid to discuss it or admit to a problem due to fear of repercussions (e.g. withdrawal of services, increased supervision and re-referral to a methadone clinic). As a result, some patients were concerned that non-compliance will result in negative consequences, as described earlier, and that because of this, the healthcare professional (in particular those with prescribing responsibility) hold the ‘power’ and can ‘control’ the patient. One patient described how he challenged his GP about this ‘power’ role:

a) Patients’ relationship with healthcare professional

The majority of participants reported a broadly positive relationship with healthcare professionals, although some of these noted that at times, there had been friction. As some had experienced negative relationships with healthcare professionals in the past, they were happy to have found a ‘good’ one, and those who had such a good relationship considered themselves ‘lucky’.

In contrast, distrust or dishonesty and concealment of problem alcohol use was a feature for those patients who reported negative relationships with healthcare professionals.

3) Patients’ views on service improvement

Patients described a number of factors which enabled or hindered the management of problem alcohol use in primary care:

Potential of primary care professionals

Importance of professional – patient relationship

Need for support and encouragement

Healthcare professional factors highlighted the central role of the practitioner-patient relationship, especially in primary care to facilitating and supporting patients through screening, treatment and ultimately, recovery. Patient or social factors highlighted the importance of motivation and associated intrinsic/extrinsic factors, especially the wider social context and how this can impact on the problem and its care. Finally, structural issues relating to how services are organised and delivered, and especially their flexibility, accessibility and capacity to address the issue, were highlighted.

A complete list of factors conducive to, or hindering, the management of problem alcohol use in primary care from patients’ perspective is listed in the ‘List of Patients’ Views on Service Improvement’ section.

“I speak. I don’t mind talking and explaining myself to people and being helpful, giving helpful information if needed” (Patient 3.3).

“Dr [name], he does say to me ‘How are you drinking, are you going to ease off, it is not good for you.’ I know he means good” (Patient 0.1)

“They were telling me before I gotta stay off the drink… ‘We’re not telling you to stay away from drink altogether, you could have the odd drink, try once a month, occasional drink.’” (Patient 5.3).

“You only get a one week supply of them, and you can’t take anymore after that… as soon as I stopped taking them, I went back on it again. They help you to sleep and they stop you from shaking from the withdrawals over the drink” (Patient 0.1).

*“I’ve done a lot of counselling over the years… it’s made me see a lot of things that I probably wouldn’t have thought about that much… It kind of gives you a chance to step back a little bit and have a look at yourself”* (Patient 17.3).

*“people are talking and they can’t wait to go home to have a can of beer. The other people that was there in the group – they are only fooling themselves… it started 10.00 in the morning.. I went in one day and there was this chap sitting beside me – the smell of drink off him was unreal”* (Patient 0.1).

“I’d no problem telling him things, there’s no point in lying to a doctor. He’s there to help you so…I was up front with him and whatever he asked me about I just told him the truth because it’s for my own benefit” (Patient 7.3).

“*I find it a bit hard… I kind of think that they don’t know what I’m going through… I don’t think you have a mind to tell you the truth, especially when you’re drinking you feel like an idiot talking about it because you’re only telling a load of lies” (Patient 5.3).*

*“Ah no, now I take it on board* [advice]*, I’m glad he has that concern” (Patient 11.3).*

“I did have arguments with him saying “You don’t realise how much power you have over people. And you are not judge, jury and executioner.” Because they have that much power over you the doctors when you’re on the methadone, you have to comply with them” (Patient 11.3).

*“he* [GP] *is not only intuitively good, but he attends very well - there is no doubt in my mind, primary care matters very much in treatment of this sort” (Patient 6.4).*

*“…because you used drugs once you will never be trusted by a doctor. Like, I’m not able to give a urine sample with somebody else in a cubicle. I just can’t… I am nearly 47, I’m an old man” (Patient 5.4)*.

*“when I used to go to counsellors it used to be just, you know, they’d give you an address, you’d go there and it’s be just like a little office, you’d go in and sit down and do your stuff. But the community places, you know, the drop in centre side of it makes it easier for people to go in”* (Patient 17.3).

*“I do think there should be decent facilities for people that are on drink you know”* (Patient 9.2).

### Summary of main themes and sub-themes

I. Patients’ experience of (knowledge, and attitudes towards) problem alcohol use

1. Patients’ use of alcohol and drinking patterns

1.1 Alcohol use history

1.1 Alcohol use and drug use

1. Attitudes to problem alcohol use

1.1 Attitudes to problem alcohol use in general

1.1 Attitudes to their own alcohol use

1. Experience and knowledge of alcohol related harm

1.1 Physical harm

1.1 Social and psychological harm

1. Knowledge of and attitudes towards safe drinking

1.1 Source of knowledge

1.1 Level of knowledge

1.1 Attitudes to safe drinking levels

II. Patients’ experience of (and attitudes towards) therapeutic interventions

5. *Experience* of therapeutic interventions for problem alcohol use

5.1 Experience of being screened for problem alcohol use

5.1 Experience of interventions for problem alcohol use

5. *Patients’ attitudes* to therapeutic interventions

5.1 Acceptance

5.1 Negative reactions

5.1 Patients’ *relationship* with healthcare professional

5.2 Positive

5.2 Negative

5. Patients views on service *improvement* (see section below)

5.1 Healthcare professional factors

5.1 Patient/Social factors

5.1 Structural factors.

### Patients’ views on service improvement

Factors conducive to, or hindering, the management of problem alcohol use in primary care from patients’ perspective:

Healthcare professional factors:

➢Potential of primary care professionals

➢Importance of professional – patient relationship

➢Need for support and encouragement

Patient factors:

➢Attitude, motivation and readiness to change

➢Motivating factors

➢family and friends

➢fear of extreme health conditions and death

➢children and family

➢self-motivation

➢Personal/social complications

➢Need to access help

Social factors:

➢Presence or absence of support and encouragement

➢supportive social environments

➢Pro-social lives

➢difficulty of adjusting after prison

➢Children and families

➢Social acceptance of alcohol

Structural factors:

➢Service delivery

➢(in) flexibility and (in)accessibility of services

➢professionals should screen opportunistically for problem alcohol use and increase supervision of ‘positive’ patients (i.e. restrictions)

➢professionals need more time to address the issue of problem alcohol use

➢Service availability

➢difficulty of attending services that do exist due to other commitments

➢need for alcohol specific services

➢need for outreach and community based services

➢ambivalent attitude towards pharmacological treatments.

## Discussion

We presented results of the first qualitative study to explore the experience of, and attitude towards, screening and treatment for problem alcohol use among problem drug users attending primary care and their views on service development. While most patients reported being screened for problem alcohol use at initial assessment, few recalled routine screening or treatment. Among the barriers and enablers to screening and treatment, patients highlighted the importance of the practitioner-patient relationship in helping them address the issue. This is embedded in the broad context of screening, treatment and patients’ attitudes towards these therapeutic interventions.

We recognise several limitations. The interviewer could have influenced the findings of the interviews by her/his interviewing style and skills, including verbal and non-verbal communication. For example, providing nods or affirmations could have prompted some interviewees to elaborate more/less on the question. Our small sample size and purpose limit the overall applicability of findings to other settings or countries. Are the results applicable to all problem drug users and not just patients on methadone treatment? Would they be applicable to other patients outside the region under study or in other countries? We cannot confirm this, but such broad generalizability is not a goal of qualitative inquiry [33]. Instead, qualitative inquiry often aims for exploration of unknown areas and generation of hypothesis for further quantitative research. In this respect, we reached our objective by formulating a hypothesis for future research as follows: ‘screening and treatment for problem alcohol use among problem drug users in primary care are both feasible and effective in primary care’.

To highlight the key strengths of our research, to our best knowledge, there is no other study published on this topic elsewhere. Several measures have been applied to increase the validity of the qualitative analysis (e.g. adopting formal approach to analysis, double-coding of pilot interviews by two independent coders, external audit by a third reviewer), and the representativeness of the sample (e.g. stratified sampling framework, random selection of practices who expressed an interest to participate). In addition, we used a standardised measure of alcohol problems to establish level of drinking among participants, which further strengthened validity and triangulation of qualitative data. Though small, our sample is broadly reflective of other cohorts attending general practice for addiction treatment.

Our results are broadly consistent with the few studies examining the topic from a perspective of general patient population. In particular, they contest the prevailing myths relating to the ‘fear of antagonizing patients over a sensitive personal issue’ and ‘alcohol being not a matter that needs to be addressed in primary health care’, as challenged by previous projects [34]. Patients felt that healthcare professionals should be more proactive in the management of problem alcohol use at a primary care level and that primary care can play an important role in their treatment [21,25].

What is new about this research is that, similar to secondary care settings, problem drug users in primary care perceive the important role of the relationship with helping professions and see stigmatising attitudes – of professionals and society – as impacting considerably on their treatment and recovery [26,28,37].

Future research should test the main hypothesis posed by this qualitative study - screening and treatment for problem alcohol use among problem drug users in primary care are both feasible and effective. A robust study design should be utilised in this evaluation (e.g. randomised trial), ideally preceded by a feasibility study to estimate key parameters for the future definitive trial. The same applies for conducting future studies in other sub-groups of problem drug users or other vulnerable populations (e.g. people with co-morbid mental health disorders). By highlighting the key role of the practitioner – patient relationship in helping problem drug users address their alcohol use, this study also highlights the importance of education and training of healthcare professionals in problem substance use and of such training incorporating issues such as complexity/co-morbidity and attitudes [38].

This research suggests that patients on methadone treatment welcome alcohol screening and intervention and perceive it as beneficial for their care in primary care settings. With a 35% prevalence of problem alcohol use among patients on methadone treatment and addiction related care increasingly being provided in European primary care, a strategic response to this issue is needed. Implementing screening and brief intervention in primary care is a priority.

## Conclusion

Problem alcohol use is an important challenge in the care of problem drug users. While primary care is well placed to address this issue, little data has reported on this. This qualitative study highlights that for patients, attending primary care for methadone treatment in Ireland’s Eastern region, screening and brief intervention are part of routine clinical practice, but should be more systematically implemented. While some patients are resistant, many are supportive of a more systematic approach to screening and brief intervention. Practitioner, patient/social and structural factors influence the practice of routine screening and brief intervention in clinical practice. The development of interventions which promote screening and brief interventions in practice are likely to benefit this at-risk group and further research and education, that help achieve this goal, are a priority. Strategies such as dissemination of clinical guidelines, educational videos, academic detailing and practice visits, should be explored.

## Endnotes

^a^Centrally held statutory register of all GPs who prescribe, pharmacists who dispense, and patients who receive methadone treatment.

## Competing interests

The authors declare that they have no competing interests.

## Authors’ contributions

WC conceived of the study, and participated in its design and coordination and helped to draft the manuscript. JK, WC, CAF conducted interviews, analysed transcripts and/or audited analysis and prepared this manuscript. WC and GB are Principal Investigator, and Co-investigator, respectively. All authors read and approved the final manuscript.

## Pre-publication history

The pre-publication history for this paper can be accessed here:

http://www.biomedcentral.com/1471-2296/14/98/prepub
